# Miniature Inverted-Repeat Transposable Elements (MITEs) in the Two Lepidopteran Genomes of *Helicoverpa armigera* and *Helicoverpa zea*

**DOI:** 10.3390/insects13040313

**Published:** 2022-03-23

**Authors:** Khouloud Klai, Marwa Zidi, Benoît Chénais, Françoise Denis, Aurore Caruso, Nathalie Casse, Maha Mezghani Khemakhem

**Affiliations:** 1 Laboratory of Biochemistry and Biotechnology (LR01ES05), Faculty of Sciences of Tunis, University of Tunis El Manar, Tunis 1068, Tunisia; khouloud.klai@fst.utm.tn (K.K.); marwa.zidi@fst.utm.tn (M.Z.); 2 Laboratory of Biology of Organisms, Stress, Health, Environment (BiOSSE), Le Mans University, 72085 Le Mans, France; bchenais@univ-lemans.fr (B.C.); fdenis@univ-lemans.fr (F.D.); aurore.caruso@univ-lemans.fr (A.C.)

**Keywords:** miniature inverted-repeats transposable elements, *Helicoverpa armigera*, *Helicoverpa zea*, insecticide resistance

## Abstract

**Simple Summary:**

Miniature inverted-repeat transposable elements (MITEs) are non-autonomous transposable elements that play important roles in genome organization and evolution. *Helicoverpa armigera* and *Helicoverpa zea* shows a high number of reported cases of insecticide resistance worldwide, having evolved resistance against pyrethroids, organophosphates, carbamates, organochlorines, and recently to macrocyclic lactone spinosad and several *Bacillus thuringiensis* toxins. In the present study, we conducted a genome screening of MITEs in the *H. armigera* and *H. zea* genomes using bioinformatics approaches, and the results revealed a total of 3570 and 7405 MITE sequences in the *H. armigera* and *H. zea* genomes, respectively. Among these MITEs, we highlighted eleven MITE insertions in the *H. armigera* defensome genes and only one MITE insertion in those of *H. zea*.

**Abstract:**

Miniature inverted-repeat transposable elements MITEs are ubiquitous, non-autonomous class II transposable elements. The moths, *Helicoverpa armigera* and *Helicoverpa zea*, are recognized as the two most serious pest species within the genus. Moreover, these pests have the ability to develop insecticide resistance. In the present study, we conducted a genome-wide analysis of MITEs present in *H. armigera* and *H. zea* genomes using the bioinformatics tool, MITE tracker. Overall, 3570 and 7405 MITE sequences were identified in *H. armigera* and *H. zea* genomes, respectively. Comparative analysis of identified MITE sequences in the two genomes led to the identification of 18 families, comprising 140 MITE members in *H. armigera* and 161 MITE members in *H. zea.* Based on target site duplication (TSD) sequences, the identified families were classified into three superfamilies (PIF/harbinger, Tc1/mariner and CACTA). Copy numbers varied from 6 to 469 for each MITE family. Finally, the analysis of MITE insertion sites in defensome genes showed intronic insertions of 11 MITEs in the cytochrome P450, ATP-binding cassette transporter (ABC) and esterase genes in *H. armigera* whereas for *H. zea*, only one MITE was retrieved in the ABC-C2 gene. These insertions could thus be involved in the insecticide resistance observed in these pests.

## 1. Introduction

Transposable elements (TEs) can play key innovating roles in their host genomes [1]. Indeed, their capacity to amplify in genomes and their ability to create new genetic variability by insertion/excision make them a rich source of genomic variants that can be selected through evolution [2,3]. Although most TE insertions are presumably deleterious or neutral, some insertions are expected to be beneficial to their carriers [4].

Miniature inverted-repeat transposable elements (MITEs) constitute a group of non-autonomous class II transposons, widespread and abundant in eukaryotic genomes [5]. MITEs are characterized by a small size (≤800 bp), a high copy number and a lack of any coding capacity [6]. They are further divided into sub-groups based on the similarity of their terminal inverted repeats (TIRs) and target site duplications (TSDs) to those of autonomous class II transposons [6]. MITEs were first described in plant genomes [7] and later found in a wide range of organisms, including insects [5,8]. It has been shown that MITEs are significant components in several insect genomes and that major MITE superfamilies are Tc1/mariner, PIF/harbinger, hAT, Mutator, and CACTA [9]. The small size of MITEs would allow them to escape the defense system of the host genome leading, therefore, to their accumulation [10]. Most MITE insertions, in multiple organisms, have been found in close proximity or even within genes [11]. Such a location entails the possibility of a functional impact on the nearby gene [12]. These results suggested that MITEs might have great effects on gene regulation and genome evolution. For example, the insertion of a MITE 200 bp upstream of the P450 gene CYP9M10 has been correlated to pyrethroid resistance in *Culex quinquefasciatus* [13].

The moths, *Helicoverpa armigera* and *Helicoverpa zea*, are both recognized as the most serious lepidopteran pests within the genus [14]. These two species diverged around 1.5 million years ago [15] and are considered sibling species due to their high morphological similarity, their genetic proximity, their emission of the same pheromone compounds but in different concentrations, and their capacity of interspecific crosses under natural and controlled environmental conditions [16]. 

*H. armigera*, the cotton bollworm, is native to the Old World (Asia, Europe, Africa, and Australasia) and is one of the most important pests worldwide. It is a polyphagous agricultural pest, and it was reported in more than 180 cultivated and wild plants, encompassing about 45 plant families [17].

*H. zea*, the corn earworm, has a wide distribution in the Americas. It is located from Canada to the south of Argentina. This species is polyphagous, and its larvae have been identified affecting leaves and fruits in more than 100 plant species [18].

*H. armigera* and *H. zea* have exhibited reduced susceptibility to groups of insecticides, including carbamates, organophosphates, pyrethroids, and *Bacillus thuringiensis* proteins [19]. Potential changes in the susceptibility of these species to conventional insecticides represent a major threat to agriculture in areas with established populations of these species and their potential hybrids. The *H. armigera* and *H. zea* genomes were sequenced in 2017 [15]. In the *H. zea* genome, there is a single strong signature of introgression around a region containing a novel, chimeric gene implicated in insecticide resistance (CYP337B3 gene), which was previously only documented in *H. armigera* and identified in the invasive population in Brazil [20]. Both *H. armigera* and *H. zea* genomes have been poorly investigated regarding their TE content. However, a previous study showed that 49% of all annotated TEs in the genome of *H. armigera* are MITEs [21]. Concerning *H. zea*, a genome walking study targeted on environment-adaptation genes showed MITEs present within or in close proximity to xenobiotic-metabolizing cytochrome P450 genes [22]. 

Here, we report the characterization of all the different groups of MITEs in the genomes of *H. armigera* and *H. zea*, using in silico approaches. The aim of this study was to provide an accurate description and comparison of MITEs in the two genomes, and to explore insecticide resistance genes in their proximity. 

## 2. Materials and Methods

### 2.1. Identification of MITEs in H. zea 

Both *H. zea* and *H. armigera* genomes were sequenced by Pearce et al. (2017) using the same technologies, from pupae of laboratory colonies derived from material collected 20 years ago [15]. The genomes were assembled by the same team using AllPaths version. JAN-2012 method. The quality of the two assemblies was tested using the QUAST tool [23] (Figures S1 and S2).

The *H. zea* genome available in GenBank-NCBI (BioProject PRJNA378438) is 341,147 Mb. This genome is assembled in 27,984 contigs and 2975 scaffolds corresponding to 20,602 kb and 201,477 kb N50 in length, respectively [15]. The *H. zea* assembled genomic sequences were downloaded and submitted to the MITE tracker tool with default parameters [6]. To find MITE candidates, MITE Tracker first searches for valid inverted repeat sequences of a given length (between 50 and 800 bp). At this step, a nucleotide−nucleotide BLAST search is used to align each MITE candidate to its reverse complement sequence (MITE maximum length was designed at 800 bp and TIRs at 10 bp). In the second step, putative MITEs were aligned and clustered into families by Vsearch [24] based on TSD and TIR sequences. Vsearch is executed with -id 0.8 (a similarity of 80% for clustering). The analysis of the obtained features allows a classification of MITE sequences into different superfamilies. For each element, right- and left-flanking sequences (sequences surrounding the element outside the TSD, by default 50 nucleotides length) are retrieved and compared with the flanking sequences of all other elements from the same family using a local alignment algorithm. The family is conserved only if the number of different individuals is equal or above a user-defined minimum copy number threshold (i.e., 3 by default). Finally, identified MITEs in *H. zea* and MITE sequences of *H. armigera* from previous study [21] were searched against GenBank, Repbase and i-MITE databases to find homologous MITEs from other insect species [9,25,26].

The evolutionary dynamics of MITEs was analyzed using the “TE” package implemented in R and based on statistical models [27]. This package estimates the age distribution based on mismatch values reflecting the deletion rate and random mutations in the sequences.

### 2.2. MITE Sequences Comparison in the H. armigera and H. zea Genomes

All annotated MITEs in *H. armigera* and *H. zea* were compared using BLAST searches with thresholds of 80% alignment length and 80% identity. The obtained MITE sequences were classified into families and superfamilies, using the 80:80:80 similarity rule and according their TSD sequences [28]. 

The identified MITE sequences in the two genomes were compared to each other and clustered based on a sequence identity higher than 80% and a minimum alignment coverage of 80% to their longest sequence. The obtained lineages were visualized by Cytoscape 3.7.2 software [29]. Additionally, the clustered MITE sequences were aligned based on a MUSCLE sequence alignment and a phylogenetic tree was generated using the maximum-likelihood method with 500 replications of bootstrap in MEGAX software [30].

### 2.3. Search for MITEs in Defensome Genes

The MITE sequences from the *H. armigera* and the *H. zea* genomes have been extended by 50 kb both upstream and downstream of their nucleotide sequences. The nucleotide BLAST was used to find defensome genes in the extended regions.

## 3. Results

### 3.1. MITEs Identification in the H. armigera and H. zea Genomes

In a previous study, we have reported on 3570 MITE sequences corresponding to seven known superfamilies and divided into 333 families in the *H. armigera* genome [21]. In the present study, MITE annotation in the genome of *H. zea* led to the identification of 7405 MITE sequences grouped in seven superfamilies and 435 families (Table 1).

Each MITE family was characterized by a short length, ranging from 50 to 800 bp and from 51 to 796 bp in *H. armigera* and *H. zea*, respectively. The TIR sequences were 10 to 32 bp long for *H. armigera* and 10 to 84 pb for *H. zea*. The Tc1/mariner was the main and the most diversified superfamily representing 1817 sequences belonging to 142 families in *H. armigera.* For *H. zea*, the main superfamily was PIF/harbinger (4587 sequences) and the most diversified family was TC1/mariner superfamily (188 families). CACTA, PiggyBac, hAT, Transib and Mavercik were the less represented superfamilies in the two analysed genomes.

Blast analysis of all MITEs identified in the two lepidopteran genomes against the Repbase and iMITE databases showed that *H. armigera* carries only one MITE which has a high similarity (83%) with a PIF/harbinger MITE from *Bombyx mori.* For *H. zea*, also only one MITE having an identity of 85% with a PIF/harbinger MITE from *Spodoptera frugiperda* was identified.

The MITE dynamic was evaluated, and the age distribution of these MITEs showed an expansion of MITE sequences at ∼90 million years ago (Mya) in *H. armigera* and a peak at 30 Mya in *H. zea* (Figure S3).

### 3.2. Characterization of MITE Families in H. armigera and H. zea Genomes

**Figure 1:**
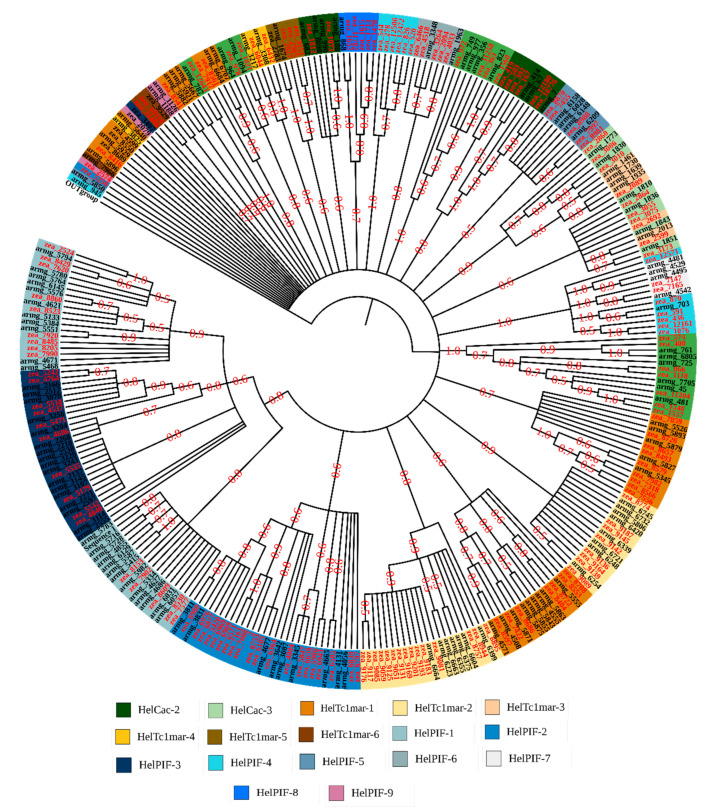
Phylogenetic analysis of MITE families from *H. armigera* and *H. zea.* The analysis was performed using the identified MITE family members having 80:80 coverage. The 18 MITE families were separated into different clades by a maximum likelihood phylogenetic analysis (model HKY85) with 500 bootstrap replications (only bootstrap value ≥ 0.5 are showed). The families belonging to the three MITE superfamilies, TC1/mariner, PIF harbinger and CACTA, are indicated in different colors and MITE members from *H. armigera H. zea* are indicated in black and red, respectively.

Comparative analysis of MITE sequences revealed a high identity between 140 MITEs (1501 copies) in *H. armigera* and 161 MITEs (1832 copies) in *H. zea* (Table 2, File S1). Based on their TIRs and TSD, we successfully identified 18 families belonging to three known superfamilies: Tc1/mariner, PIF/harbinger and CACTA. We named the PIF/harbinger families as *Helicoverpa* PIF/harbinger (HelPIF) 1 to 9, the Tc1/mariner families as *Helicoverpa* Tc1/mariner (HelTc1mar) one to six and the CACTA families as *Helicoverpa* CACTA (HelCac) one to three (Table 2). The clustering analysis of these MITEs using the cytoscape tool showed the separation of the 18 groups. The MITE sequences from *H. armigera* and *H. zea* belonging to the same family were grouped into the same cluster (Figure S4). These clusters were retrieved in the phylogenetic analysis with some difference for some families (Figure 1). For the PIF/harbinger superfamily, the results showed a clear separation of MITE members belonging to seven families, except for HelPIF-4 and HelPIF-7, showing a high sequence variation between MITE members. The MITEs of some TC1/mariner families appeared dispersed, in particular, the HelTc1mar-1 family split into five clades and the HelTc1mar-3 family clustered with the HelCac-3 family. MITEs belonging to the HelCac-1 and HelCac-2 are divided in four and two clades, respectively. However, all the 18 families showed a clear and a strong link between *H. armigera* and *H. zea* MITEs.

Eleven MITE families (HelPIF-1, -2, -5, -7, -9, HelTc1mar-1, -3, -4 and HelCac-1, -2, -3) were similar in copy numbers between both *Helicoverpa* genomes but only 7 MITE families showed 2- to 6-fold differences in total sequence number (underlined in red in Table 2). The HelPIF-2 family displays the highest copy numbers with 469 and 324 copies in *H. armigera* and *H. zea*, respectively, whereas the HelTc1mar-6 and HelTc1mar-4 families have the fewest copies corresponding to 6 and 10 copies in *H. armigera* and *H. zea*, respectively (Table 2). 

### 3.3. MITE Insertions Analysis in Relation with Defensome Genes

BLAST searches led to the identification of 11 MITE insertions in defensome genes in the *H. armigera* genome. The TSD classification of the involved MITEs revealed that these sequences are members of Tc1/mariner and CACTA superfamilies (Table 3). Further analysis of MITE insertions has shown that six MITEs were inserted in cytochrome P450 genes, four elements were retrieved in ATP binding cassette transporter subfamily G gene and five were hosted by esterase genes. All inserted MITEs have intronic insertion sites.

For *H. zea,* we successfully identified 20 defensome genes by similarity with *H. armigera* (Table S1). We identified only one MITE sequence inserted in an ATP binding cassette transporter subfamily C2 (ABCC2) gene. This MITE was 137 bp long and belonged to the PIF/harbinger superfamily (Table 3). 

Three MITE sequences were previously identified in the genome of *H. zea* and were inserted in the cytochrome P450 gene CYP9A14 (GenBank accession DQ788840.1) [22]. Blast searches with the CYP9A14 gene sequence identified the genomic scaffold KZ117493.1 (35,331 bp in length) in the public *H. zea* genome assembly (80% identity and 65% query cover). The gene position in this scaffold was between 13,600 bp and 16,000 bp, and only one MITE sequence, located from 17,076 bp to 17,206 bp, was identified upstream the gene. This MITE was 130 bp and belonged to the TC1/mariner superfamily (Table 3).

## 4. Discussion

In this study, we performed a systematic analysis to identify MITE families in the *H. armigera* and *H. zea* genomes published in year 2017 and publicly available. These genomes were both obtained from the pupae of laboratory colonies, by the same team, with the same technologies of sequencing and assembling, with a scaffold assembly level and comparable quality. The identification of MITE sequences was performed using a computational tool, MITE-tracker based on TIRs and TSDs features. In a previous study, a total of 3570 MITE sequences were identified in the genome of *H. armigera* [21]. In the present analysis, results showed a 2-fold difference in total MITE sequences in *H. zea* genome (7405 sequences) versus *H. armigera* genome. From all identified MITEs in *H. armigera*, only few MITEs were retrieved in the new genome assembly of *H. armigera* larvae available on NCBI from March 2021 (ASM1716586v1). This difference can be due to the molecular (difference in sequencing technology), biological (larvae vs. pupae) and bioinformatics difference between the two sequenced genomes. 

The evolutionary dynamics and age distribution of identified MITEs in the *H. armigera* and *H. zea* genomes revealed a more recent and important burst of these sequences in *H. zea* genome at 30 Mya. However, it should be noted that the evolutionary dynamics and transposition timing may not be informative for very short and degenerated sequences such MITEs. This may partly explain the difference between the present expansion times and the divergence time of the two species, estimated to be 1.5 Mya according to the mitochondrial genome analysis performed by Behere et al. (2017) [31]. In addition, this difference may also be explained by a reactivation or invasion of active elements (by horizontal transfer) whose intact protein would interact with TIRs of MITEs and trigger their expansion. However, a more detailed analysis would be necessary to confirm this hypothesis.

The TSD classification of annotated MITEs showed that in *H. armigera* the main MITE superfamily was the Tc1/mariner superfamily, which is one of the predominant transposable element superfamilies in *H. armigera* genome [21] but in *H. zea*, the main MITE superfamily was the PIF/harbinger superfamily suggesting important genomic differences between the two sibling species.

In previous study, two MITE sequences (HzMITE2-1 and HzMITE3) were identified in a laboratory strain and a midgut cell line RP-HzGUT-AW1 of *H. zea* [22] which were retrieved in the present study and in the sequenced genome with less than 40% identity and cover.

The total MITE sequences identified in the two *Helicoverpa* genomes is much higher than the data from Han et al. (2016) in which the MITE hunter tool identified only 726 MITE sequences in nine lepidopteran species. This confirms the interest of the MITE tracker tool to increase the detection of MITEs in insect genomes, as it appears to be more efficient in detecting MITEs compared to other currently available tools.

Several studies tried to identify MITEs in insect genomes. Recently, 84 MITE families covering 1.3 Mb were identified in the genome of the diptera *Mayetiola destructor* [32]. In the hemidipteran *Bemisia tabaci* genome, 71 MITE sequences were identified as *mariner-like element* derivatives [33]. Eight novel families of MITEs were discovered in the African malaria mosquito, *Anopheles gambiae* [5]. In *Bombyx mori*, 17 MITE families with a total of 5785 members were identified [8]. However, MITE sequences available in databases are very restricted. The database of transposable elements, Repbase, contains only seven insect MITE sequences from *Aedes aegypti* [25] and the iMITE database include 6012 MITE sequences from 98 insect genomes [9]. Blast analysis of MITEs against Repbase and iMITE databases have shown a high similarity for only two MITEs of *H. armigera* and *H. zea* with two known MITEs in *Bombyx mori* and *Spodoptera frugiperda,* suggesting that the two lepidopteran genomes contain a high number of new or unknown MITE sequences. This may also be due to the lack of significant sequence conservation between MITEs of divergent species.

The identified MITE sequences were divided into 333 and 435 families in the *H. armigera* and *H. zea* genome, respectively, suggesting a tremendous diversity in the *H. armigera* and *H. zea* MITEs. The TIRs and TSDs similarity of MITE sequences inside different families suggests that they may arise from the amplification of a few progenitor copies. After blast searches and manual editing, a total of 18 MITE families were identified in *H. armigera* and *H. zea* genomes. Cytoscape and phylogenetic analysis showed that the MITE sequences in both genomes were closely related and showed a high identity level between MITEs of the two genomes. MITE members from certain family showed a dispersion into different clades, particularly the HelTc1mar-1 family. This dispersion may be due to the small size of the MITEs and great care must be taken in interpreting the phylogenetic analysis of such sequences without their original TEs. 

The numbers of MITEs in 12 families were similar, suggesting that the major sequences of these families evolved before the divergence of the two species around 1.5 Mya. However, seven MITE families (HelPIF-3, 4, 6, 8, HelTc1mar-2, 5, 6) displayed large variation in copy numbers between the two species. The MITE families examined herein were classified into three superfamilies based on their TSDs, which were the main MITE superfamilies in the *H. armigera* and *H. zea* genomes. 

After searching for defensome genes, eleven MITEs were found inserted in cytochrome P450, ABC transporter and esterase defensome genes of *H. armigera*. All inserted MITEs are located in intron. They could result in exon skipping, alternative splicing, or even in alterations in expression profiles if the corresponding introns contain regulatory sequences as exemplified by the Mu insertion into an intron of the knotted locus in maize [34].

In P450 genes, six MITEs were identified in *H. armigera*. The overexpression of P450 genes has been reported to increase the ability to metabolize insecticides in insect pests with agricultural importance and public health [35]. In *Aedes aegypti*, the P450 4C1 was highly upregulated in a resistant strain [36]. Three MITEs were inserted in carboxylesterase genes in *H. armigera*. Temephos resistance has been associated with the up-regulation, through gene amplification, of two carboxylesterase (CCE) genes closely linked on the genome of *Aedes albopictus* [37]. In the ABCG member-20 gene of *H. armigera*, a single MITE was identified in the position 89,853–90,098 bp. In a previous study, a hAT transposon in the position 1486–2436 bp and a Tc1/mariner transposon from 91,998 bp to 93,071 bp were inserted in this *ABCG20* gene [21] indicating that several types of TE can be inserted into this *ABCG20* gene. It is likely that each type of TEs may induce different genomic changes upon transposition. Consequently, this ABC gene should be gnomically highly variable. 

For *H. zea*, only one MITE belonging to the PIF/harbinger superfamily was inserted in an *ABCC2* gene. The function of this *ABCC2* gene was recently evaluated in *H. zea*, using CRISPR/Cas9 and results observed support that this gene is not a major Cry1Ac receptor in this insect [38].

Thus, the present study will deepen our knowledge of MITEs and their position near defensome genes, which are fundamental to understanding the evolution and adaptation of these pest genomes and insecticides resistance of these species.

## 5. Conclusions

Genomic analysis of *H. zea* identified 7405 MITE sequences belonging to the Tc1/mariner, PIF harbinger, CACTA, PiggyBac, hAT, Transib and Maverick superfamilies. The genome-wide comparative study of around 300 MITE members belonging to 18 families in *H. armigera* and *H. zea* genomes highlighted important genomic similarities between the two sibling species. At all, 11 MITEs were identified in defensome genes in *H. armigera* and only one MITE in the genome of *H. zea*. This disparity is mainly due to the poor annotation of *H. zea* genome, for which, in particular, a very small number of defensome genes have been identified. The identification and characterization of MITEs in the two lepidopteran genomes provide a basis for further studies on their genomic impact. It will also facilitate the use of MITE insertions to identify genetic factors associated with defensome genes of *H. armigera* and *H zea.*

## Figures and Tables

**Table 1:** MITE superfamilies detected in *H. armigera* and *H. zea* genomes.

		*H. armigera* *				*H. zea*			
Superfamilies	TSD	MITEs Length (pb)	TIR Length (pb)	MITE Sequences	Families	MITEs Length (pb)	TIR Length (pb)	MITE Sequences	Families
Tc1/mariner	TA	50–360	10–21	1817	142	85–794	10–71	2328	188
PIF/Harbinger	TWA, AT or AWT	55–685	15–32	1368	111	60–720	13–84	4587	154
CACTA	2–4 bp	78–775	10–26	250	70	51–796	17–49	450	86
PiggyBac	TTAA	50–800	15–31	93	2	256–775	21–69	25	4
hAT	8 bp	56–260	17–29	20	3	120–550	28–63	15	1
Transib	5 pb	83–386	13–27	16	2	75–564	36–57	5	1
Maverick	6 pb	50–800	10–24	6	3	306–654	18–42	5	1
**Total**				**3570**	**333**			**7415**	**435**

(*) Values from our previous study [21].

**Table 2:** Characteristics of the 18 MITE families in the *H. armigera* and *H. zea* genomes.

				*H. armigera*		*H. zea*	
	Families	MITE Length (bp)	TIR Length (bp)	Sequences Number	Copies Number	MITE Sequences	Copies Number
1	HelPIF-1	155–234	25–65	25	210	13	174
2	HelPIF-2	121–160	22–38	17	469	11	324
3	HelPIF-3	213–229	58–72	9	161	21	323
4	HelPIF-4	374–439	30–43	2	27	11	53
5	HelPIF-5	111–140	35–44	5	73	5	81
6	HelPIF-6	198–256	53–69	2	15	5	31
7	HelPIF-7	163–174	39–47	4	47	2	39
8	HelPIF-8	367–385	26–36	1	7	5	21
9	HelPIF-9	290–339	22–37	2	21	2	15
	**Total PIF/Harbinger**	**111**–**439**	**22**–**72**	**67**	**1030**	**75**	**1061**
10	HelTc1mar-1	129–164	21–57	23	114	27	173
11	HelTc1mar-2	98–125	28–38	14	103	20	205
12	HelTc1mar-3	255–295	16–24	5	55	2	76
13	HelTc1mar-4	195–212	21–34	3	18	2	10
14	HelTc1mar-5	260–283	37–59	1	8	4	48
15	HelTc1mar-6	457–679	10–29	1	6	3	36
	**Total Tc1/mariner**	**98**–**679**	**10**–**59**	**47**	**304**	**58**	**548**
16	HelCac-1	348–469	16–25	13	76	13	81
17	HelCac-2	352–486	16–46	7	40	8	67
18	HelCac-3	260–274	15–20	6	51	7	75
	**Total CACTA**	**260**–**486**	**15**–**46**	**26**	**167**	**28**	**223**
	**Total MITEs**			**140**	**1501**	**161**	**1832**

**Table 3:** MITE insertions in defensome genes of *H. armigera* and *H. zea* genomes.

	Gene Families	Gene Name	Gene Length	MITE Inserted Name	MITE Length (bp)	TIR Length (bp)	Insertion Position	Exon/Intron Position
***Helicoverpa armigera***	Cytochrome P450	CYP450 4V2-like (LOC110375933)	14,709	MITE_armg_527	390	18	9793–10,182	Intron 11
		CYP450 4V2-like (LOC110375407)	8843	MITE_armg_2987	189	57	8301–8489	Intron 11
		CYP450 4C1-like (LOC110375947)	10,768	MITE_armg_4229	202	56	3478–3651	Intron 4
		CYP450 6B5-like (LOC110371743)	15,792	MITE_armg_5115	129	27	3727–3855	Intron 1
		Probable CYP450 49a1 (LOC110372238)	38,869	MITE_armg_6331	107	17	8631–8737	Intron 2
		Probable CYP450 6d2 (LOC110383081)	4170	MITE_armg_6757	96	26	1309–1404	Intron 1
	ABC	ABCG 49-like (LOC110377844)	48,302	MITE_armg_7483	486	21	25,885–26,370	Intron 1
		ABCG 49-like (LOC110374586)	55,698	MITE_armg_5600	136	21	30,248–30,383	Intron 23
		ABCG 20 (LOC110376033)	96,146	MITE_armg_1805	246	10	89,853–90,098	Intron 12
		ABCG 23 (LOC110373590)	60,734	MITE_armg_5758	124	32	855–978	Intron 1
	Esterase	Esterase FE4 like (LOC110380254)	9333	MITE_armg_1387	287	13	3323–3609	Intron 4
		Esterase FE4 like (LOC110384365)	8458	MITE_armg_6918	641	17	2829–3469	Intron 1
		Carboxylesterase 1C (LOC110375169)	6830	MITE_armg_5488	140	144	6127–6266	Intron 10
		Carboxylesterase 1E (LOC110379202)	27,073	MITE_armg_1116	334	50	18,270–18,603	Intron 3
		Venom carboxylesterase-6 (LOC110373494)	27,998	MITE_armg_335	393	40	9211–9603	Intron 1
***Helicoverpa zea***	ABC transporter	ABC C2 (KY701524.1)	11560	MITE_zea_8125	137	15	2663–2799	Intron 5
	Cytochrome P450	CYP9A14 (KZ117493.1)	2400	MITE_zea_5100	130	10	-	Upstream

## Data Availability

Not applicable.

## Supplementary Materials

The following supporting information can be downloaded at: https://www.mdpi.com/article/10.3390/insects13040313/s1. File S1. MITE sequences of 18 families in *H. armigera* and *H. zea* genomes. Table S1. Defensome gene identification in *H. zea* genome by similarity with *H. armigera*. Table S2. Mismatch calculation of identified MITEs in *H. armigera*. Table S3. Mismatch calculation of identified MITEs in *H. zea*, Figure S1. Cumulative length in *H.armigera* and *H. zea* assemblies. Figure S2. GC content in the *H. armigera* and *H. zea* genomes. Figure S3. Age distribution of MITEs in *H.* *armigera* (A) and *H. zea* (B). Figure S4. Cytoscape visualisation of MITE families belonging to PIF-harbinger (A), Tc1/mariner (B) and CACTA (C) superfamily in *H. armigera* and *H. zea* genomes.
