# Supplementary material for: Miniature Inverted-Repeat Transposable Elements (MITEs) in the Two Lepidopteran Genomes of Helicoverpa armigera and Helicoverpa zea
Source: Insects. 2022 Mar 23;13(4):313. doi: 10.3390/insects13040313 (PMC9033116; doi:10.3390/insects13040313)
Supplement: Supplementary file 1 [file insects-13-00313-s001.zip › Figures S1-S4.pdf]

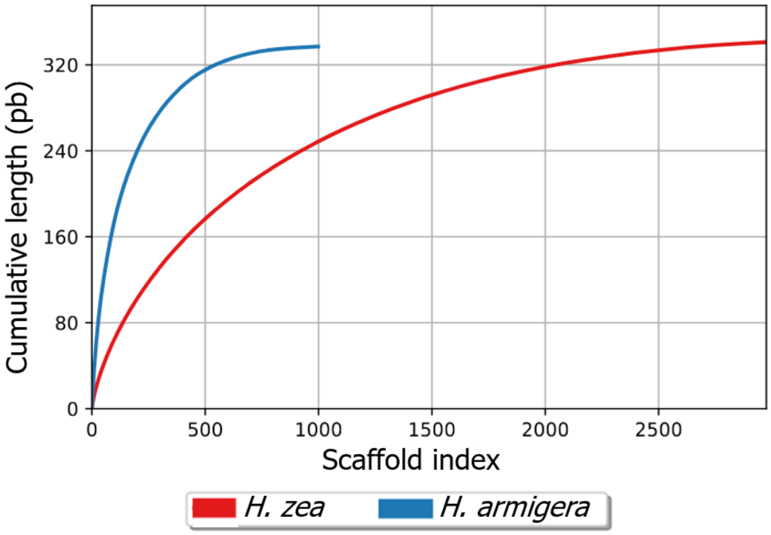

Figure S1. Cumulative length in *H. armigera* and *H. zea* assemblies

Scaffolds are ordered from largest to smallest (in number of bases), the plot shows the number of bases in the first *x* scaffold, as *x* varies from zero to the number of scaffolds.

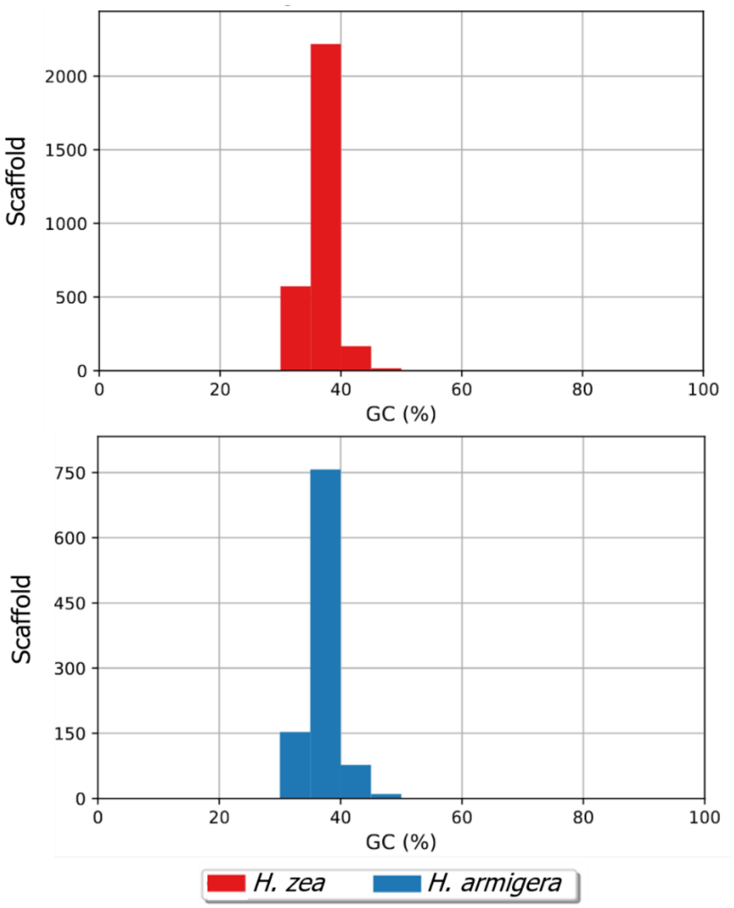

Figure S2. GC content in the *H. armigera* and *H. zea* genomes

These histograms show the distribution of GC content in the scaffolds of *H. armigera* and *H. zea* genomes. The *x* value shows the per cent of GC (from 0 to 100). The *y* value shows the number of scaffolds whose GC content is x.

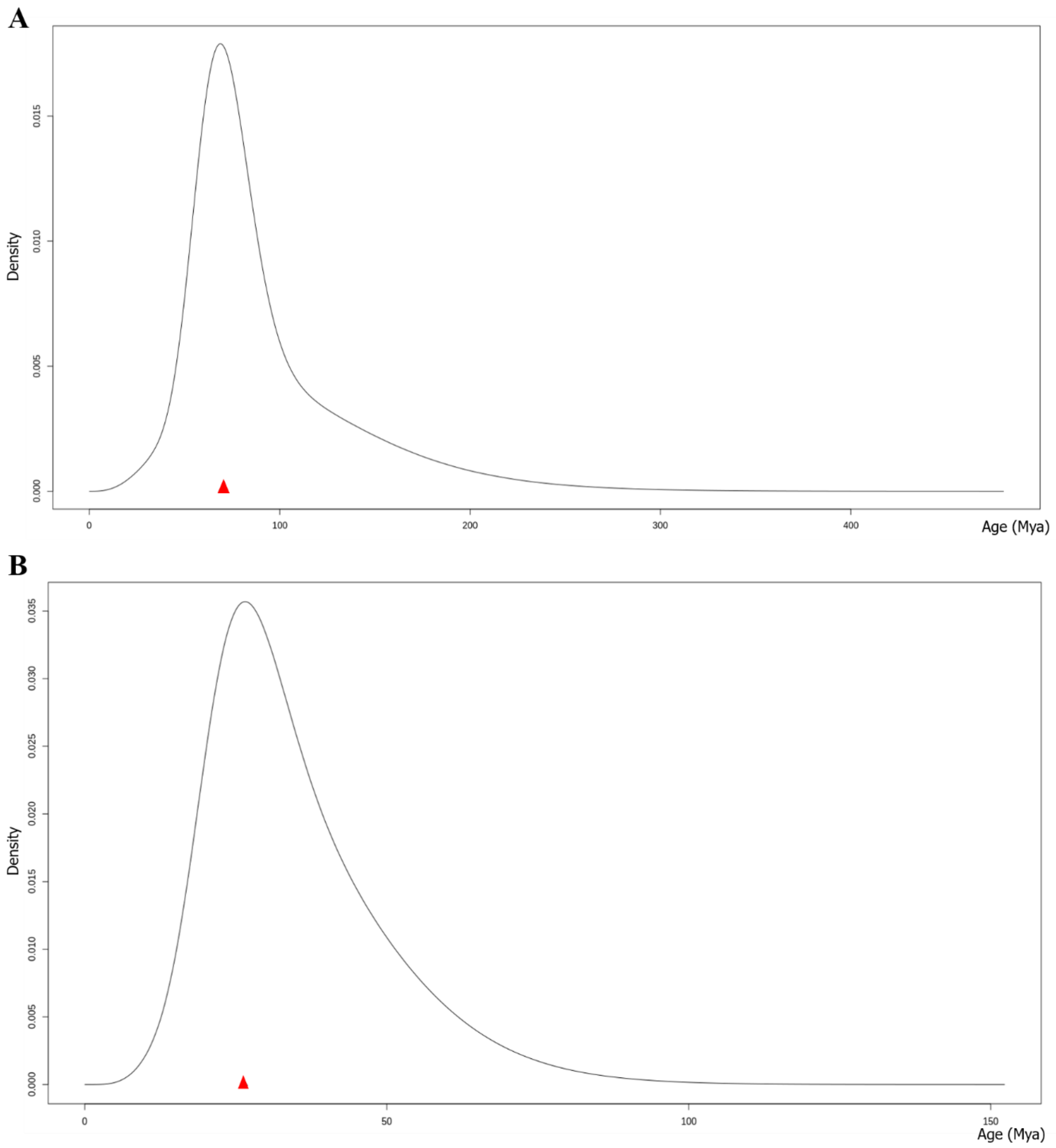

Figure S3. Age distribution of MITEs in *H. armigera* (A) and *H. zea* (B)

These graphs were generated using "TE" package implemented in R (Dai et al., 2018), the graph A shows the age distribution of MITEs in *H. armigera* and the B graph shows this of *H. zea*. In the two graphs, the *x* value shows the age in Mya (Million years ago). The *y* value shows the MITEs density.

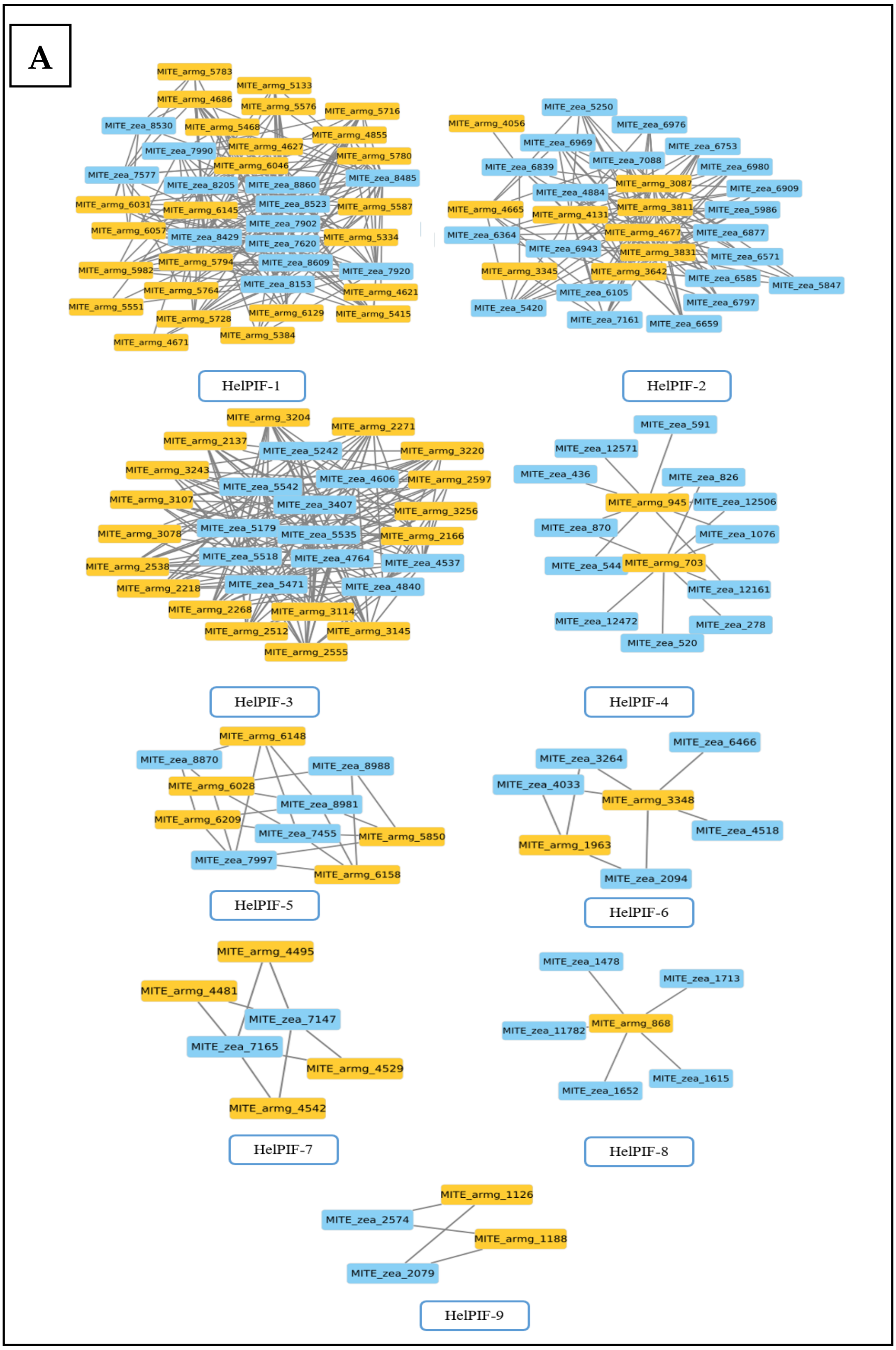

A
MITE_armg_5783
MITE_armg_5133
MITE_armg_4686
MITE_armg_5576
MITE_armg_5716
MITE_zea_8530
MITE_armg_5468
MITE_armg_4855
MITE_zea_7990
MITE_armg_4627
MITE_armg_5780
MITE_zea_7577
MITE_armg_6046
MITE_zea_8485
MITE_zea_8205
MITE_zea_8860
MITE_armg_6031
MITE_armg_6145
MITE_zea_8523
MITE_armg_5587
MITE_zea_7902
MITE_armg_6057
MITE_zea_8429
MITE_armg_5334
MITE_zea_7620
MITE_armg_5794
MITE_zea_8609
MITE_armg_5982
MITE_zea_7920
MITE_armg_5764
MITE_zea_8153
MITE_armg_4621
MITE_armg_5551
MITE_armg_5728
MITE_armg_6129
MITE_armg_5415
MITE_armg_5384
MITE_armg_4671
HelPIF-1
MITE_zea_5250
MITE_armg_4056
MITE_zea_6976
MITE_zea_6969
MITE_zea_6753
MITE_zea_6839
MITE_zea_7088
MITE_zea_6980
MITE_armg_3087
MITE_zea_4884
MITE_zea_6909
MITE_armg_4665
MITE_armg_3811
MITE_armg_4131
MITE_zea_5986
MITE_zea_6364
MITE_armg_4677
MITE_zea_6877
MITE_zea_6943
MITE_armg_3831
MITE_zea_6571
MITE_armg_3345
MITE_armg_3642
MITE_zea_6585
MITE_zea_5847
MITE_zea_6105
MITE_zea_6797
MITE_zea_5420
MITE_zea_7161
MITE_zea_6659
HelPIF-2
MITE_armg_3204
MITE_armg_2271
MITE_armg_2137
MITE_zea_5242
MITE_armg_3220
MITE_armg_3243
MITE_zea_5542
MITE_zea_4606
MITE_armg_2597
MITE_armg_3107
MITE_zea_3407
MITE_armg_3256
MITE_armg_3078
MITE_zea_5179
MITE_zea_5535
MITE_armg_2166
MITE_zea_5518
MITE_zea_4764
MITE_armg_2538
MITE_zea_4537
MITE_zea_5471
MITE_armg_2218
MITE_zea_4840
MITE_armg_2268
MITE_armg_3114
MITE_armg_2512
MITE_armg_3145
MITE_armg_2555
HelPIF-3
MITE_zea_591
MITE_zea_12571
MITE_zea_436
MITE_zea_826
MITE_armg_945
MITE_zea_12506
MITE_zea_870
MITE_zea_1076
MITE_zea_544
MITE_armg_703
MITE_zea_12161
MITE_zea_12472
MITE_zea_278
MITE_zea_520
HelPIF-4
MITE_armg_6148
MITE_zea_8870
MITE_zea_8988
MITE_armg_6028
MITE_zea_8981
MITE_armg_6209
MITE_zea_7455
MITE_armg_5850
MITE_zea_7997
MITE_armg_6158
HelPIF-5
MITE_zea_6466
MITE_zea_3264
MITE_zea_4033
MITE_armg_3348
MITE_zea_4518
MITE_armg_1963
MITE_zea_2094
HelPIF-6
MITE_armg_4495
MITE_armg_4481
MITE_zea_7147
MITE_zea_7165
MITE_armg_4529
MITE_armg_4542
HelPIF-7
MITE_zea_1478
MITE_zea_1713
MITE_zea_11782
MITE_armg_868
MITE_zea_1615
MITE_zea_1652
HelPIF-8
MITE_armg_1126
MITE_zea_2574
MITE_armg_1188
MITE_zea_2079
HelPIF-9

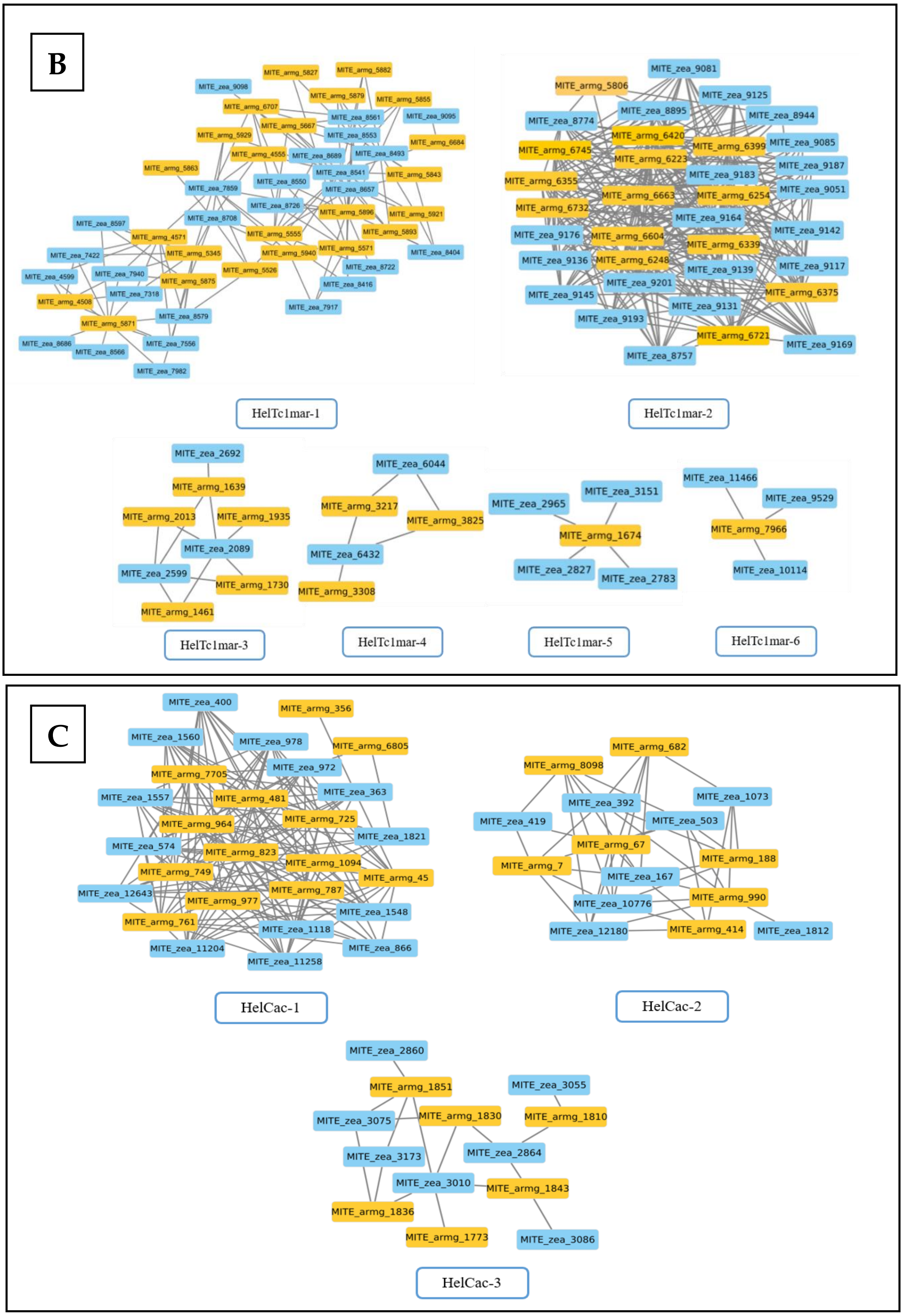

Figure S4. Cytoscape visualisation of MITE families belonging to PIF-harbinger (A), Tc1/mariner (B) and CACTA (C) superfamily in *H. armigera* and *H. zea* genomes.
This analysis was performed using Cytoscape tool. The blue boxes refer to *H. zea* MITEs while the yellow refer to *H. armigera* MITEs and the lines represent the link between MITE sequences.
